# Gender Patterns in Immigrants’ Health Profiles in France: Tobacco, Alcohol, Obesity and Self-Reported Health

**DOI:** 10.3390/ijerph17238759

**Published:** 2020-11-25

**Authors:** Myriam Khlat, Stéphane Legleye, Damien Bricard

**Affiliations:** 1 Institut National d’Etudes Démographiques (Ined), 93300 Aubervilliers, France; khlat@ined.fr (M.K.); bricard@irdes.fr (D.B.); 2 Institut National de la Statistique et des Etudes Economiques (INSEE), 92120 Montrouge, France; 3 Inserm U1178, 75014 Paris, France; 4 Institut de Recherche et Documentation en Economie de la Santé (IRDES), 75019 Paris, France

**Keywords:** smoking, alcohol, obesity, self-reported health, gender, immigrants, France

## Abstract

*Background:* to date, little attention has been given to gender differences in the health of migrants relative to native-born. In this study, we examine the health profile of the largest immigrant groups in metropolitan France, considering several health indicators and with a special interest in the gendered patterns. *Methods*: The data originate from the 2017 Health Barometer survey representative of metropolitan France. A subsample of 19,857 individuals aged 18–70 years was analysed using modified Poisson regression, and risk ratio estimates (RR) were provided for the different migrant groups regarding alcohol use, current smoking, obesity and less-than-good self-reported health, adjusting for age and educational level. *Results*: None of the groups of male migrants differs from the native-born in terms of self-reported health, and they have healthier behaviours for alcohol (men from sub-Saharan Africa: 0.42 (0.29–0.61)) and from the Maghreb: 0.30 (0.1–0.54)) and smoking (men from sub-Saharan Africa: 0.64 (0.4–0.84)), with less frequent obesity (men from the Maghreb: 0.61 (0.3–0.95)). The latter, however, more frequently report current smoking (1.21 (1.0–1.46)). For women, less-than-good health is more frequently reported by the groups from sub-Saharan Africa (1.42 (1.1–1.75)) and from the Maghreb (1.55 (1.3–1.84)). Healthier behaviours were found for alcohol (women from overseas *départements*: 0.38 (0.1–0.85)) and from the Maghreb: (0.18 (0.0–0.57)) and current smoking (women from southern Europe: 0.68 (0.4–0.97), from sub-Saharan Africa: 0.23 (0.1–0.38) and from the Maghreb: 0.42 (0.2–0.61)). Conversely, some were more frequently obese (women from overseas *départements*: 1.79 (1.2–2.56) and from sub-Saharan Africa: 1.67 (1.2–2.23)). In the latter two groups from Africa, there is a larger relative male excess for tobacco than in the native-born (male-to-female ratios of respectively 2.87 (1.6–5.09) and 3.1 (2.0–4.65) vs 1.13 (1.0–1.20)) and there is a female excess for obesity (0.51 (0.2–0.89) and 0.41 (0.2–0.67)) in contrast with the native-born (1.07 (0.9–1.16)). The female disadvantage in terms of less-than-good self-reported health is more pronounced among migrants from the Maghreb than among the natives (0.56(0.4–0.46) vs. 0.86 (0.8–0.91)). *Conclusion*: Considering a set of four health indicators, we provide evidence for distinctive gender patterns among immigrants in France. Male immigrants have a healthy behavioural profile in comparison with the natives and no health disadvantage. Female immigrants have a more mixed profile, with a health disadvantage for the non-Western groups from Africa. The contribution to this discordance of socioeconomic factors and gender relations needs to be investigated.

## 1. Introduction

The “healthy migrant effect” and “unhealthy assimilation” hypotheses are dominant paradigms in the field of migrant health, reflecting the progressive deterioration over time of the initially superior health of migrants. Those paradigms have not been analysed with gender lens [1], which means that they are gender-blind, i.e., that they do not differentiate between male and female migrants. Yet, there are good reasons to believe that there could be gender-specific health profiles and variations over time, as men and women have different life experiences and social determinants in both origin and destination countries. Analysing the role of gender in determining migrants’ health profile is important both from a theoretical viewpoint for a better conceptualization of the migrants’ health framework and from a policy viewpoint to develop appropriate preventive actions to preserve the health capital of migrants.

Health differences between men and women reflect in part the inequalities related to the gender roles, likely to be wider in some of the societies of origin of the immigrants. To start with, women in developing countries are more socially disadvantaged relative to men than women in developed countries. They indeed have lower employment rates, lower salaries and have to take charge of the bulk of childcare and domestic tasks. Although gender roles are likely to be renegotiated after migration, male and female migrants have very different employment trajectories, as the latter are directed to unskilled and caretaking services, with precarious job contracts and long working hours, in addition to a heavy load of domestic duties.

The more proximal health determinants such as the health behaviours are influenced by social norms in the country of origin and assumed to converge with those of the destination country. There are also in this respect distinctive gender patterns. For instance, female immigrants from certain cultures may retain low alcohol and tobacco intake and distinctive reproductive patterns which may protect them from the major cancers, but those favourable features may be accompanied by unfavourable changes in terms of nutritional and cardiovascular risk factors [2,3]. On the other hand, male immigrants from certain groups may increase their tobacco consumption to a much greater extent than female immigrants [4]. Many reasons may explain the adoption of unhealthy behaviours, among which the attempt to cope with discrimination-related stress [5]. Overall, the behavioural dynamics is considered to be responsible in part for the erosion of the health advantage over time [6], although some of those changes are beneficial to health whereas others are detrimental.

Yet, insufficient attention has been given in the international literature to gender differences in health behaviours and differential behavioural changes in immigrant populations in relation with the “unhealthy assimilation hypothesis”. Second, as the differences between migrants and native-born vary across health indicators, and as, above that, there are also distinctive gender patterns for different indicators, some authors have concluded that “presenting results on a single factor in isolation could lead to a misinterpretation of associations” [7]. Generally, health behaviours tend to be more favourable among immigrants than self-reported health, particularly at older ages [8]. A comprehensive view looking at different indicators simultaneously would provide a better vision of the specific health patterns of immigrants.

There is a wealth of studies on migrants’ health in Europe but few provide comparisons with the native-born, and there is a large variability in design and methodology. In Spain, immigrants displayed better lifestyle-related parameters, with lower consumption of alcohol and tobacco than the autochtonous population, but consumption increased with duration of stay [9,10]. In France, male immigrants from Northern Africa tended to have higher smoking levels, as opposed to females from the same region, whose consumption was particularly low [11]. In a literature review on acculturation, obesity and health behaviours among immigrants to high-income countries, which included six studies from Europe, acculturation was found to be associated with weight gain among immigrants, but there were inconsistencies in findings according to gender [12]. For self-reported health, another study found support for the healthy immigrant hypothesis among both men and women in Western Europe [13], but worse health was reported for immigrants aged 50 years and above in a set of 11 European countries [8].

In certain studies, gender was treated as a confounder (see for instance [8,9]). In other studies, although stratified analyses were conducted, the interpretation of the findings did not integrate the specificities of gender roles among immigrants in the host country environment. In addition, most of those studies focused on specific health behaviours, eventually along with self-reported health but with no attempt to highlight larger profiles considering different health indicators.

France offers excellent potential for exploring smoking disparities by geographical origin, given the diversity and importance of the groups present in the country. In 2014, the share of the immigrant population in France approached 9% (https://www.insee.fr/fr/statistiques/1410693). Our aim is to contribute to a better understanding of those insufficiently explored dimensions of gender patterns and health profiles of male and female immigrants, using data from metropolitan France.

Considering different migrant groups, our research questions are: (1) How do male and female migrants fare in terms of self-reported health in comparison with the native-born? (i.e., to what extent is there evidence in support of the “healthy migrant effect” vs. the “unhealthy assimilation” paradigm), and; (2) What are the specific behavioural profiles of male and female migrants and how do they relate to their health profiles?

We examine alcohol and tobacco use, corpulence and self-reported health in the largest immigrant groups originating from North Africa, sub-Saharan Africa, Southern Europe and French overseas departments. Our analytical approach involves analysis of the prevalence levels of the different conditions in immigrants relative to the native-born, separating men and women and adjusting on the main sociodemographic factors. In addition, we estimate male-to-female prevalence ratios in the different groups for the various health indicators. Our interpretation builds on this comprehensive set of statistics to describe the specific health profiles of the immigrant groups and their specific gender patterns relative to those of the native-born.

## 2. Method

### 2.1. Data

The 2017 “Health Barometer” is a nationwide French survey that used a two-stage random (household/individual) sample and a dual sampling frame (landline/mobile line) to measure health perceptions and behaviours of the general population [14]. This investigation was approved by the French Commission on Individual Data Protection and Public Liberties (the Commission Nationale Informatique et Libertés (CNIL)), and all data collected were anonymous and self-reported. The response rate was 48.5%. The number of telephone numbers (landlines and mobile) available to join the selected person within the household was used to compute pseudosampling weights; final weights were obtained through a calibration procedure considering gender x age (in 5 categories), number of household members, educational level, size of the urban area and region of residence, in order to match the distribution of the last national Labor Force Survey. The initial sample of respondents comprised 25,319 individuals aged 18–75 years having answered questions about their geographical origin, health behaviours (tobacco, alcohol, weight and height) and self-reported health. Our analyses concerned the subsample of 22,208 individuals aged 18–75 years and born either in metropolitan France (excluding the second generations) or in any of the geographical regions of interest. The detailed methodology can be found in [15].

### 2.2. Measures

Daily tobacco smoking was defined as a current smoking of tobacco (whatever the type of product), excluding the electronic cigarette; regular alcohol use was defined as the report of at least 3 days of alcohol use per week in the last 12 months. Obesity was defined as a body mass index (BMI) over 30 based on reported weight and height; less than-good self-reported health as the report of a correct/poor/very poor health.

Immigrants were categorized based on six items of information: place of birth and nationality at birth and mother’s and father’s place of birth and nationality. Immigrants from North Africa were subdivided into repatriates (French nationals born in the former colonies of North Africa) and natives from those colonies born without the French nationality, who are referred to hereinafter as the Maghrebins. The latter term (Maghrebins) is derived from an Arabic term for the North African region of origin, to refer to immigrants and their descendants who have neither European backgrounds nor Jewish origins [16]. The analysis was limited to the following reference group: born French in metropolitan France from parents born in metropolitan France; born in overseas *départements*; born in Southern Europe (Italy, Spain, Portugal, Greece); Maghrebins; born in sub-Saharan Africa (Table 1).

As education is a major determinant of health behaviours [17], we considered the highest level of education as a control variable. For comparative purposes across countries, education was categorized in four levels, based on national typologies using ISCED (International Standard Classification of Education) standards [18]: (1) ISCED 0, 1 and 2 levels: below upper secondary education (Low); (2) ISCED 3 and 4: upper secondary education and postsecondary non tertiary education (Medium); (3) ISCED 5: short-cycle tertiary education (High-short); (4) ISCED 6 and over: at least a Bachelor’s degree or equivalent level (High-long). The educational gradient in prevalence for the different indicators was quantified using the relative index of inequality [19] that allow comparisons of populations with different education levels and age, taking into account the change in values of highest level of education across time and place. The ridit, a rank variable of our 4-category variable of education for each gender [20], was computed for this purpose. Ridit were estimated by gender and age group (18–39, 40–59, 60–75). The relative index of inequality (RII) is the risk-ratio of the ridit, which contrasts the prevalence of the least to the most educated in the population, taking into account the distribution of education in the population. RII estimates lower than 1 correspond to higher prevalence among the most educated (positive gradient), whereas RII estimates greater than 1 correspond to lower prevalence among the most educated (negative gradient).

### 2.3. Statistical Analyses

Multivariate dichotomous modified Poisson regressions [21] modelling the prevalence were run, adjusting for group of origin, ridit, age and age-squared. The regressions were conducted separately for men and women, except for the estimation of the male-to-female prevalence ratio. Additional regressions further adjusted for four other factors: occupational situation (currently working or not), subjective financial situation (at ease, just, difficult), having lost one’s job in the last 12 months or fearing to lose one’s job, household type. All analyses were conducted with SAS 9.4.

## 3. Results

### 3.1. Sociodemographic Profiles (Table 1)

Immigrants from Southern Europe belong to the oldest waves and stand out as being the older group (median ages of respectively 52 and 53 years), with the longest median duration of stay of all immigrant groups (46 years for both genders), and the smallest median age at migration (12 years for men and 15 years for women). The latter statistic indicates that a large proportion arrived as children either accompanying their parents or for family reunification. This group as a whole is also the least educated, but the gap may be partly explained by a cohort effect in a context of rising educational levels over time in all countries.

The two groups of immigrants from Overseas “Départements” and from the Maghreb are younger than the native-born (median ages in the early forties) and stand close to each other with respect to their median duration of stay and to their age at arrival, ranging around 20 years. Both have larger proportions of lower educated than the natives, with however a smaller gap for women from overseas *départements* (27% with low education vs. 24%).

As immigrants from sub-Saharan Africa belong to the most recent migratory wave represented in the study, they are the youngest group (median ages of respectively 41 years and 39 years for men and women), and their median duration of stay is relatively short (respectively 15 and 14 years). Their median age at migration is the highest of all groups (24 years for men and 21 years for women), reflecting the importance of labour migration. Overall, immigrants from sub-Saharan Africa are much less educated than the native-born (38% in men and 37% in females were in the lowest educational levels vs. 20% and 24%).

### 3.2. Health Profiles 

For each of the four health indicators, we conducted two series of stratified multivariate analyses in order to investigate: (1) the modifying effect of gender on migration-related disparities, and; (2) the modifying effect of migration background on gender disparities.

**Migration-related disparities for men and for women:** this analysis examines the extent to which male and female immigrants are different in the way they compare to their native-born counterparts (upper part of Table 2). 

In the group of immigrants from overseas *départements*, males were similar to the native-born in all indicators considered, whereas females had a much lower regular alcohol use (0.38 (0.17–0.85)) and, at the opposite, a much higher obesity prevalence than the native-born (1.79 (1.25–2.56)). 

In the group of immigrants from Southern Europe, males were similar in all respects to the reference population, unlike females, whose current smoking was significantly less frequent than that of the native-born (0.68 (0.47–0.97)).

In the group of immigrants from sub-Saharan Africa, males had healthier behaviours than the native-born with respect to alcohol (0.42 (0.29–0.61)) and tobacco consumption (0.64 (0.49–0.84)). Females had an even greater advantage than males for tobacco (0.23 (0.14–0.38)), along with significant disadvantages in terms of obesity (1.67 (1.25–2.23)), and less-than-good self-reported health (1.42 (1.15–1.75)). 

In the group of immigrants from the Maghreb, men and women both had much lower prevalence of regular alcohol use (respectively 0.30 (0.16–0.54) and 0.18 (0.06–0.57)), while they had opposite patterns for tobacco, as current smoking was slightly more prevalent in men (1.21 (1.01–1.46)), and much less in females (0.42 (0.29–0.61)). Differences were also observed for obesity, as men had a specific advantage in this respect (0.61 (0.39–0.95)) and for less-than-good self-reported health, with a significant disadvantage for women (1.55 (1.30–1.84)).

**Gender disparities in immigrant groups and in natives**: this analysis examines the male-to-female prevalence ratios in the different groups and compares the gender contrast for the different indicators to what is observed among the natives (bottom part of Table 2).

Among the native-born, the male-to-female prevalence ratio was balanced only for obesity, whereas men had a much higher prevalence of regular alcohol use (3.18 (2.93–3.45)) and a higher level of current smoking (1.13 (1.07–1.20)). Although their behaviours were less healthy, men were less likely than females to rate their health as less-than-good (0.86 (0.81–0.91)). 

The gender distortion for less-than-good self-reported health was also found among immigrants from sub-Saharan Africa (0.65 (0.47–0.90)), and, to a much greater extent than among the natives, in the group from the Maghreb (0.56 (0.41–0.76)). For regular alcohol use, all immigrants except those coming from sub-Saharan Africa showed higher male-to-female risk ratios than the native born (although none of the differences were significant). On the opposite, the immigrants from sub-Saharan Africa showed a more gender-balanced ratio with RR = 1.64 (0.89–3.03) that was almost significantly lower than the ratio observed in the natives (*p* = 0.064). The gender relative difference for current smoking among immigrants from overseas *départements* (1.68 (1.04–2.71)) and among those from Southern Europe (1.60 (1.06–2.42)) was not significantly different from that found for the natives, while the gap was significantly larger for the groups from Africa (sub-Saharans 2.87 (1.61–5.09) and Maghrebins 3.10 (2.06–4.65)). The latter two groups were also the only groups for which there was a significant gender contrast for obesity, in favour of males (0.41 (0.25–0.67) for immigrants from sub-Saharan Africa and 0.51 (0.29–0.89) for immigrants from the Maghreb). Finally, immigrants from the Maghreb tended also to have lower gender-ratios than the natives for the less-than-good health (0.56 (0.41-0.76) vs. 0.86 (0.81–0.91) respectively).

## 4. Discussion

The originality of our study lies in the combination of a health profile approach with a gender perspective. First, the joint consideration of different health indicators gives much depth to the analysis, as it allows identification of the distinctive assets and vulnerabilities of the groups in presence. Further to that, the separate analysis of men and women reveals very specific patterns of departure from the native-born in the two genders.

Theoretically, the evidence can be interpreted within the framework of the so-called “healthy immigrant effect” and “unhealthy assimilation” hypotheses, and we propose to integrate the gender perspective to more fully interpret the findings. In a review on health-related lifestyles among immigrants in Europe, Spadea et al. [22] conclude that longer durations of residence were found in the literature to be associated with more unhealthy behaviours, and particularly weight gain, and that unhealthy behaviours were ”exacerbated by the sense of loneliness and low levels of integration in the host country”. Our data are cross-sectional, with no retrospective health information allowing an exploration of changes over the lifetime, and particularly of changes after arrival. Variation by duration of residence could have been explored. Duration effects are however subject to confounding by cohort effects, and, in addition, the majority of migrants have arrived for more than 10 years (76% of migrants from overseas *départements*, 89% of those from Southern Europe, 65% of those from sub-Saharan Africa and 78% of those from the Maghreb).

Non-Western male immigrants reported healthier behaviours than the native-born: for alcohol and tobacco in those from sub-Saharan Africa, and for alcohol and obesity for the Maghrebins, whose advantage was however mitigated by a significantly elevated tobacco consumption. Some female immigrants also tended to consume less alcohol (those from the Maghreb and from French overseas *départements*) and less tobacco (from Southern Europe, sub-Saharan Africa and the Maghreb) than the native-born. However, those favourable behaviours were partly offset by a higher prevalence of obesity (for those from French overseas *départements* and those from sub-Saharan Africa). The major difference between the two genders was that the two groups of non-Western female immigrants, i.e., those from sub-Saharan Africa and from the Maghreb, were the only groups with a significantly higher prevalence of less-than-good self-reported health than the native-born. This is all the more striking for females from the Maghreb, who would be expected to reap the health benefits of their very moderate tobacco and alcohol consumption.

Those gender-specific migration-related disparities are reflected in the distinctive gender patterns which we find for the two groups of non-Western immigrants. For immigrants, the gender pattern may reflect partly the situation in the country of origin, but it may also depend on the differential acculturation processes in the two genders. Among the native-born, the two indicators for which there is a significant difference between men and women go in opposite directions, with a more than three-fold elevated prevalence of regular alcohol use in men relative to women, as opposed to a 15% lower prevalence of less-than-good self-reported health. The two immigrant groups which depart from this pattern are those from Africa, in which there is a larger relative male excess for tobacco, a larger relative female excess for obesity and for less-than-good self-reported health (although not significantly so for the sub-Saharans).

Our finding of both a higher smoking prevalence among Maghrebin men and a lower prevalence among sub-Saharan men in comparison with the native-born, as well as much lower levels for the females from the two groups accord with reports from earlier studies based on French national health surveys from the late eighties [23], the early nineties [24] and on the Baromètre Santé 2010 survey [4,11]. The specific patterns of those two groups of immigrants may be related to the relative position of their regions of origin with respect to the smoking epidemic dynamics, as north African countries were positioned in the years 2000 in stage 2, with increasing levels of smoking, while sub-Saharan countries were still in stage 1, i.e., at low levels [25]. In a literature review of studies on smoking in immigrants from non-western to western countries, Reiss et al. [26] highlight the common feature of high prevalence among less acculturated men and low prevalence among less acculturated women originating from non-western countries. As the smoking male-to-female prevalence ratio was found to be strongly correlated worldwide with the gender empowerment measure [27], the impressive gap found in this study supports the idea of the persistence of traditional gender norms long after immigration. In particular, the situation for Maghrebins may be interpreted as reflecting the enduring influence of gendered norms postmigration.

In a recent review of acculturation, obesity and health behaviours among immigrants to high-income countries [12], there was extensive evidence for weight gain among people migrating from low/middle-income to high-income countries, with an association between acculturation (measured with standard measures or as duration of stay) and obesity. The recommendation issued by the authors was that interventions should be promoted at the initial stages following migration to avoid uptake of unhealthy behaviours. However, there was no attention in this review on eventual gendered patterns of weight gain. In our study, obesity stands out as a distinctive feature for female immigrants from two regions, the French overseas départements and sub-Saharan Africa, with consequently a very large gender gap in favour of males, as opposed to a balanced ratio among the native-born. It should be noted that those populations share common ethnic and socioeconomic features, as the population from French overseas *départements* (Guadeloupe, Martinique, French Guiana, Reunion), which represent the remainder of the past colonial empire, is predominantly Afro-Caribbean and Creole and on the whole socioeconomically disadvantaged.

In our study, the only assessment of health properly speaking was the subjective indicator of self-reported health. As was observed for obesity, we find a gendered pattern of disadvantage, with female immigrants from both sub-Saharan Africa and the Maghreb, much more likely to report less-than-good self-reported health than the native-born. The estimate for females from overseas *départements*, although elevated, does not reach significance. For sub-Saharan females, the health disadvantage may be related to their higher prevalence of obesity, possibly associated with a range of chronic diseases (particularly diabetes) and physical or physiological limitations. As for the situation of Maghrebin females, it is paradoxical, as they have a favourable profile of health behaviours, notably for alcohol and tobacco consumption, and no disadvantage for obesity.

The absence of evidence on predictors of bad health raises question on the nature of the conditions or diseases affecting this group of women from the Maghreb. In the literature, some studies echo this finding of gender-based differential health of immigrants. A specific disadvantage of female immigrants was observed in the adult population aged 25–64 living in Catalonia, Spain, as female immigrant groups showed excess risk of poor self-perceived health, whereas male immigrant groups had better health than natives in the same social class [28]. Several explanations were advanced by the authors: (1) a greater extent of health-related selection (and consequently “health-related effect”) among men than among women; (2) wider gender inequalities and an enhancement of the submissive role of women in immigrants’ societies of origin, and; (3) the exposure of immigrant women from working classes to the “cumulative burden of socio-economic and gender-related disadvantages and disempowerments”. The latter hypothesis was further developed by Brabete [29], according to whom immigrant women are subjected to continuous stress, given the particularly heavy load in terms of housework and childcare which they have to carry after working hours. In the long run, persistent stress may lead to depression. In a recent study in Germany, immigrants reported significantly more depression, generalized anxiety and panic attacks in the past four weeks and suicidal ideation compared to nonimmigrants, but sex was used as a confounder in the analysis rather than a stratification variable [30]. More generally, there is a lack of studies on mental health of immigrants providing information on the differential situation of men and women.

## 5. Conclusions

Few empirical studies on health and health behaviours of immigrants have focused on gender-specific patterns in comparison with the native-born. Our study concerns groups of established migrants in France of whom the most recently arrived (from sub-Saharan Africa) have a median duration of stay around 15 years. We find a large amount of variation across regions of origin for both male and female migrants, as well as across genders. In terms of self-reported health, no male or female migrant group benefit from an advantage, whereas the two female groups from Africa display a significant disadvantage. In terms of behaviours, male migrants have similar or healthier behaviours than the native-born, with the exception of those from the Maghreb who are more likely to be regular smokers. Female migrants have similar or healthier behaviours than the natives, with the exception of those from overseas *départements* and those from sub-Saharan Africa, who are both characterized by a higher prevalence of obesity.

In summary, our findings do not provide evidence for either gender in support of the “healthy migrant hypothesis”. Conversely, the health situation of the two non-Western female groups (from sub-Saharan Africa and the Maghreb) is compatible with the “unhealthy assimilation” paradigm. The health disadvantage of females from sub-Saharan Africa may be related to their greater prevalence of obesity and possibly cardio-vascular risk factors. However, no explanation in terms of health behaviours emerges for females from the Maghreb. For this specific group, more traditional gender roles and the unequal division of productive and domestic work needs to be investigated.

## Figures and Tables

**Table 1:** Study sample by background characteristics (Baromètre Santé 2017 survey, ages 18–70 years).

Country/Region of Birth	*n*	Median Age at Survey (Years)	Median Age at Migration (Years)	Median Duration of Stay (Years)	Low Education (%)
MEN					
France (native-born)	9049	46.2	N.A.	N.A.	20
Overseas *départements*	118	41.4	19	20	32
Southern Europe	183	51.8	12	46	44
sub-Saharan Africa	283	41.2	24	15	38
Maghreb	229	44.1	21	18	35
WOMEN					
France (native-born)	10,808	46.8	N.A.		24
Overseas *départements*	157	43.2	19	19	27
Southern Europe	197	52.8	15	46	58
sub-Saharan Africa	274	38.9	21	14	37
Maghreb	248	43.4	20	22	41

Median and percentages are weighted.

**Table 2:** Risk ratios and male-to-female ratios for regular alcohol use, current smoking, obesity and less-than-good self-reported health (Adjusted on age, age^2^ and relative educational level (Ridit), Baromètre Santé 2017 survey, ages 18–70 years).

Country/Region of Birth	Regular Alcohol Use	Current Smoking	Obesity	Less-Than-Good Self-Reported Health
MEN				
France (native-born)	1	1	1	1
Overseas *départements*	0.64 (0.38–1.08)	1.03 (0.76–1.39)	1.14 (0.66–1.96)	1.15 (0.78–1.69)
Southern Europe	1.14 (0.89–1.46)	0.98 (0.76–1.27)	0.75 (0.47–1.18)	1.03 (0.77–1.38)
sub-Saharan Africa	**0.42** (0.29–0.61)	**0.64** (0.49–0.84)	0.76 (0.49–1.16)	1.15 (0.89–1.48)
Maghreb	**0.30** (0.16–0.54)	**1.21** (1.01–1.46)	**0.61** (**0.39**–**0.95**)	1.04 (0.79–1.36)
WOMEN				
France (native-born)	1	1	1	1
Overseas *départements*	**0.38** (0.17–0.85)	0.68 (0.46–1.01)	**1.79** (1.25–2.56)	1.24 (0.94–1.64)
Southern Europe	1.00 (0.60–1.65)	**0.68** (0.47–0.97)	1.18 (0.80–1.75)	1.00 (0.78–1.28)
sub-Saharan Africa	0.88 (0.53–1.47)	**0.23** (0.14–0.38)	**1.67** (1.25–2.23)	**1.42** (1.15–1.75)
Maghreb	**0.18** (0.06–0.57)	**0.42** (0.29–0.61)	1.16 (0.82–1.64)	**1.55** (1.30–1.84)
MALE-TO-FEMALE RATIO				
France (native-born)	**3.18** (2.93–3.45)	**1.13** (1.07–1.20)	1.07 (0.98–1.16)	**0.86** (0.81–0.91)
Overseas *départements*	**5.78** (2.31–14.42)	**1.68** (1.04–2.71)	0.67 (0.35–1.25)	0.78 (0.49–1.22)
Southern Europe	**4.05** (2.32–7.09)	**1.60** (1.06–2.42)	0.63 ^a^ (0.35–1.14)	0.89 (0.62–1.29)
sub-Saharan Africa	1.64 (0.89–3.03)	**2.87**^b^ (1.61–5.09)	**0.41**^b^ (0.25–0.67)	**0.65** (0.47–0.90)
Maghreb	**6.20** (1.66–22.88)	**3.10**^b^ (2.06–4.65)	**0.51**^a^ (0.29–0.89)	**0.56**^b^ (0.41–0.76)

Significance test of comparison of group-specific male-to-female risk ratio with reference population risk ratio: ^a^: *p* < 0.05; ^b^: *p* < 0.01; estimates in bold are significantly different from unity
